# Cytokine inhibition in chronic fatigue syndrome patients: study protocol for a randomized controlled trial

**DOI:** 10.1186/s13063-015-0971-z

**Published:** 2015-10-05

**Authors:** Megan E. Roerink, Hans Knoop, Sebastian J. H. Bredie, Michael Heijnen, Leo A. B. Joosten, Mihai G. Netea, Charles A. Dinarello, Jos W. M. van der Meer

**Affiliations:** Department of Internal Medicine, Radboud University Medical Center, Post Box 9101, 6500 HB Nijmegen, The Netherlands; Expert Centre Chronic Fatigue, Radboud University Medical Center, Nijmegen, The Netherlands

**Keywords:** Chronic fatigue syndrome, Treatment, Protocol, Anakinra, Placebo, Interleukin-1, Cytokine

## Abstract

**Background:**

Chronic fatigue syndrome (CFS) is a medically unexplained syndrome for which no somatic or pharmacological treatment has been proven effective. Dysfunction of the cytokine network has been suspected to play a role in the pathophysiology of CFS. The disturbances of the cytokine network detected in CFS patients are highly variable, in part due to the lack of adequate controls in many studies. Furthermore, all studies have been performed on peripheral venous blood of patients. As cytokines mainly act in tissues, for example, the brain, the information that can be derived from peripheral blood cells is limited. The information regarding the possible role of cytokines in the pathophysiology could come from intervention studies in which the activities of relevant cytokines are reduced, for example, reducing interleukin-1, interleukin-6 or tumor necrosis factor. In this study, the clinical usefulness of anakinra, an IL-1 antagonist, will be assessed in patients with CFS.

**Methods/Design:**

A randomized placebo-controlled, double-blind trial will be conducted. Fifty adult female patients meeting the Centers for Disease Control (CDC) criteria for CFS and without psychiatric co-morbidity will be included. After inclusion, patients will be randomized between treatment with anakinra (recombinant human interleukin-1 receptor antagonist) or placebo. Each group will be treated for 4 weeks. Outcome measures will be assessed at baseline, after 4 weeks of intervention, and 6 months after baseline assessment. The primary outcome measure will be fatigue severity at 4 weeks, measured with the validated Checklist of Individual Strength (CIS). Secondary outcome measures are functional impairment, physical and social functioning, psychological distress, pain severity, presence of accompanying symptoms, and cytokine and cortisol concentrations.

**Discussion:**

This is the first randomized placebo-controlled trial that will evaluate the effect of interference with IL-1 on the experience of fatigue in patients with CFS. The results of this study may expand treatment options for patients with CFS, for whom graded exercise therapy and cognitive behavioral therapy are the only evidence-based interventions that exist at this moment.

**Trial registration:**

Clinicaltrials.gov: NCT02108210. Clinicaltrials.gov registration date: 8 April 2014. EudraCT: 2013-005466-19

## Background

Chronic fatigue syndrome (CFS) is a medically unexplained syndrome characterized by severe disabling fatigue for a period of at least 6 months, which leads to considerable impairment in daily functioning [1]. Various accompanying symptoms may be present, such as headache, joint and muscle pain, sore throat, impaired memory and concentration and exercise intolerance. In the Netherlands, the prevalence of CFS is at least 27,000 persons [2]. So far, the cause for CFS is yet unclear [3]. Cognitive behavioral therapy (CBT) and graded exercise therapy (GET) are the only interventions that have shown positive results in randomized controlled clinical trials for treating fatigue-associated CFS symptoms and disability [4–8].

Cytokines are hormone-like proteins that convey messages between cells. Originally, they were thought to act only within the host defense system, but soon it became clear that they mediate an array of diverse effects in normal physiology and disease. Since proinflammatory cytokines play a key role in inflammation (for example, by causing fever, inducing muscle pain, fatigue, sleep and other flu like symptoms), they have been hypothesized to be responsible for the symptoms in CFS [9, 10].

Several studies have been performed to investigate whether there is an excess of cytokines in CFS, but so far, findings are inconsistent [11, 12]. A recent systematic review on circulating cytokines in CFS reported that the majority of studies performed during the past years did not find significantly increased concentrations of proinflammatory cytokines [13]. A major problem is that many studies did not use adequate controls and used different methods to handle blood samples. Cytokine responses are under genetic control, but they are extremely vulnerable to other influences, such as hormonal status, food, exercise, stress, behavior, drugs and vaccines [14]. Therefore, it is not easy to compose a good control group. An additional problem is that almost all studies have been performed on peripheral venous blood. As cytokines mainly act in tissues, with the brain being the most important target organ in CFS, information that can be derived from studying circulating cytokine concentrations (which are generally in the pg/ml range) is limited. The only information regarding a role of cytokines that is pathophysiologically relevant could come from intervention studies in which crucial cytokines in tissue are inhibited. A potentially relevant cytokine, which can be blocked in humans without severe side effects, is interleukin-1 (IL-1) [15].

Although it is plausible that cytokines play a role in the pathophysiology of CFS, there is only indirect evidence for this theory:

1. The complaints of patients with CFS are often described as that of a persistent flu. During infections like influenza, symptoms are generally ascribed to the action of cytokines (like IL-1, IL-6, tumor necrosis factor alpha (TNF) and interferons) [9].
2. Many disease states are accompanied by anorexia, loss of interest, somnolence and fatigue, a symptom complex coined as sickness behavior. The cytokines IL-1beta, TNF and IL-6 are thought to be responsible for it. Administration of either IL-1, IL-6, TNF or each of the interferons to humans and animals is accompanied by flu-like symptoms [16–18].
3. Previously, it was reported that IL-8 and IL-10 were significantly elevated in the cerebrospinal fluid in patients with CFS, compatible with induction of IL-1 [19].
4. Beta amyloid precursor protein has also been found to be elevated in the cerebrospinal fluid of CFS patients [20]. Production of this protein is under control of IL-1 and TNF [21–23].
5. Our group has previously established that patients with CFS have a significant loss of gray matter in the brain [24, 25]. This loss of gray matter might be caused by enhanced cytokine activity.

Drugs that interfere with the proinflammatory cytokine IL-1 are commonly used nowadays for a variety of inflammatory disorders [26]. The recombinant IL-1 receptor antagonist (anakinra) reduces the activity of both IL-1α and IL-1ß by binding to the IL-1 receptor. This intervention is highly targeted and hence would allow investigators to draw conclusions regarding the pathophysiology of CFS, and the effect of reducing cytokine concentrations in CFS patients. Moreover, compared to blocking TNF-α or IL-6, blocking IL-1 with anakinra has a long safety recorded with respect to side effects, and is not associated with increased susceptibility to opportunistic infections such as Mycobacterium tuberculosis.

The primary aim of this study is to assess the effect of anakinra on fatigue severity in patients with CFS. Fatigue is the most central and characterizing symptom of CFS, and in contrast to the accompanying symptoms, it is reported by all patients. It is also strongly related to the functional impairments reported by patients. As a secondary study aim, we will assess the effect of anakinra on the level of functional impairment, physical and social functioning, pain severity, presence of accompanying symptoms and psychological distress. In this paper, we describe the protocol to evaluate the effects of anakinra. Other studies with anakinra or anti-IL-1ß revealed a decrease in fatigue [27–30].

## Methods/Design

### Study design

A randomized placebo-controlled trial (RCT) will be performed to determine whether interference with IL-1 is able to reduce fatigue and disabilities in CFS patients. Within each study arm, treatment will be double blind. The study will be performed in the Radboud University Medical Center, in the Department of Internal Medicine and in the Expert Center for Chronic Fatigue (ECCF), located in Nijmegen, the Netherlands. All female CFS patients visiting the outpatient clinic of the department of Internal Medicine or visiting the ECCF will be considered for participation. Furthermore, patients connected to the “ME/CVS-stichting,” a Dutch foundation for CFS patients, will be asked to participate in the study. To increase homogeneity in our study population, we decided to only include female patients, as CFS is a disease that mostly affects women. After inclusion, each patient will receive an individual study code. Patients will be asked to bring a healthy, age-matched, neighborhood control to their first study visit. If patients decide not to participate in this study, an attempt will be made to elucidate the reason for this, but patients are not obligated to explain their refusal.

### Study population

Fifty subsequent patients will be included and equally randomized between treatment arms. Inclusion criteria for participation comprise the CDC diagnosis of CFS [20, 31], fatigue duration ≤ 10 years or recent progression of fatigue severity, female sex, age between 18 and 59 years old, a score ≥ 40 on the subscale fatigue severity of the Checklist of Individual Strength (CIS), and a score ≥ 700 on the Sickness Impact Profile (SIP). The exclusion criteria include females who are pregnant or nursing, intend to get pregnant during the study, use or have used psychotropic medication in the past month, received a live vaccine during the last 4 weeks, had substance abuse in the past 3 months, have had symptoms more than 10 years, are taking any medication except oral contraceptives and/or paracetamol, have evident somatic comorbidity, have current engagement in CFS research, do not have the ability to understand the nature and the extent of the trial and the procedure required, have psychiatric comorbidity (major depression, psychosis, eating disorders, anxiety disorders, bipolar disease and post-traumatic stress disorder) assessed with ‘The Mini-International Neuropsychiatric Interview’ (M.I.N.I.) [32], or are currently engaged in a legal procedure with respect to disability claims.

### Ethical approval

The study is approved by the Medical Ethical Review Committee of the Radboud UMC Nijmegen (registration number 2014/025). The study is registered at the European Union Drug Regulating Authorities (EudraCT: 2013–005466–19) and will be conducted according to the Declaration of Helsinki, Good Clinical Practice (GCP) and Good Manufacturing Practice (GMP) guidelines. The inclusion of patients started in July 2014. Written informed consent will be obtained from all patients before participation in the trial.

### Study medication, randomization and follow-up

**Fig. 1:**
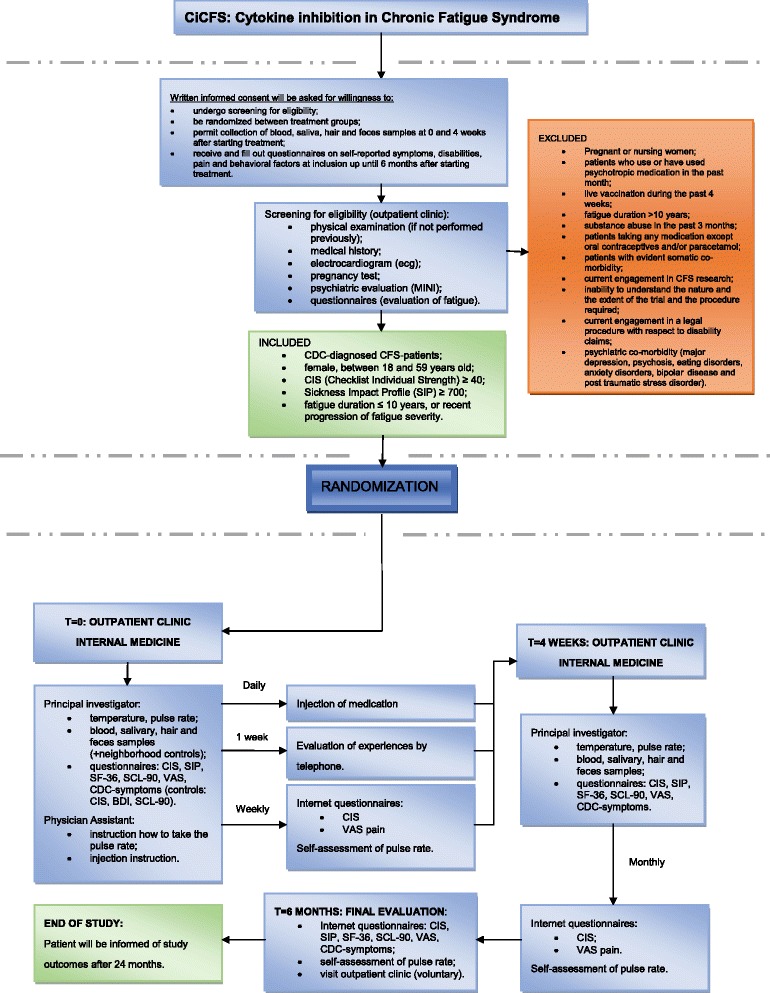
Study-specific procedures

Eligibility for participation of patients is determined at the pre-study visit. After giving informed consent, patients will be screened for inclusion and exclusion criteria by means of the examinations listed in Fig. 1. Laboratory investigations performed earlier will be evaluated for all patients and will be repeated if not performed recently, or when essential measurements, as recommended by others [31], are missing. Patients who qualify to be included will be randomized 1:1 to receive either anakinra (100 mg/day) or placebo. Randomization will be performed by the study pharmacist (Department of Clinical Pharmacology, Radboud University Medical Center), will be known only by the pharmacist and will be exposed only in case of emergency. If the code is broken, it will render the participant not eligible. When the study is completed, the randomization list will be made available by the study pharmacist.

Study medication will be provided by the Swedish Orphan Biovitrium (Sobi) and stored at the Department of Pharmacology of the RadboudUMC. Preparation and labelling of anakinra and placebo will be done according to the current guidelines. This will be performed by the Clinical Trials Unit department of Clinical Pharmacology of the RadboudUMC. The anakinra and placebo syringes will be identical in appearance; the placebo syringes will contain a mixture of sodium citrate, sodium chloride and polysorbate. Medication is used once daily, during a period of 4 weeks. The anakinra and placebo will be provided by the main investigator. On the first study day, patients will be instructed how to self-inject the study medication, as described by others [27]. Administration takes place in the subcutaneous tissue, most often the abdomen or the thighs. During the study, patients will be advised to set their alarm clock daily at the same time to remind them to use the medication correctly. Drug adherence will be evaluated after 1 week and after completion of treatment. Patients will return all used and unused syringes after 4 weeks.

During the intervention period, use of co-medication is only allowed when used for ≤ 14 consecutive days and on the condition that there are no known interactions with anakinra. Oral contraceptives and/or paracetamol can be used without limitation. During the follow-up period, there are no limitations regarding the use of medication. All co-medication will be registered and reported afterwards.

Since the anakinra arm is difficult to blind because of local reactions at the injection site, the research physician and the principal investigator will not be informed of the side effects. After the injection instruction by the physician assistant (PA), the patient will be instructed to report adverse effects to the PA and not to the research physician. The PA will report all side effects to an independent physician. The independent physician will examine patients if needed and is mandated to stop treatment. To evaluate blinding of the treatment, patients will be asked which medication they thought they were using during the trial, after they have completed the study.

Study visits are carried out at week 0, week 4 and, if needed, after 6 months. After 1 week, patients will be contacted by telephone to evaluate the occurrence of any problems regarding the use of medication and drug utilization will be recorded. If there are serious side effects, an additional study visit can be performed by the independent physician at any time. Between study visits, subjects will be asked to fill out web-based questionnaires up to 6 months after their first study visit (Fig. 1).

### Outcome measures

The primary outcome measure is fatigue severity measured with the subscale fatigue severity of the Checklist of Individual Strength (CIS) at 4 weeks, the primary endpoint of the study. Scores on the CIS subscale range from 8 to 56 (8 items, 7-point Likert Scale). This questionnaire has been validated extensively in patients with chronic fatigue syndrome [33, 34]. Patients will fill out this web-based questionnaire weekly during the course of the trial and monthly during the follow-up period (Fig. 1).

### Secondary outcome measures

Secondary outcome measures:

1. Level of functional impairment. The level of functional impairment will be measured with the Sickness Impact Profile (SIP8) total score. The SIP8 is a validated instrument to evaluate sickness-related dysfunction. The total score gives an indication of the experienced disabilities in all domains of functioning [35].
2. Physical and social functioning. Physical and social functioning will be assessed with the subscale physical functioning and subscale social functioning of the Short Form (SF)-36 [36].
3. Level of psychological distress. The level of psychological distress will be assessed with the total score on the Symptom Checklist-90 (SCL-90). A high total score reflects psychological distress [37].
4. Pain severity. Pain severity will be assessed with a visual analog scale (VAS). This score can vary between 0 (no pain) and 10 (worst pain ever experienced).
5. Presence of accompanying symptoms. The presence of accompanying symptoms will be evaluated using the CDC criteria. The number of symptoms can vary between 0 and 8.
6. Cytokine concentration in blood. The cytokine concentrations in blood (plasma and blood in Pax-gene tubes) will be determined. Our study can provide additional information regarding cytokine levels because we have the opportunity to compare cytokine concentrations with healthy neighborhood controls. Also we will compare pre-treatment concentrations with post-treatment concentrations.
7. Cortisol concentration in hair and saliva. Cortisol concentrations in hair and saliva will be assessed because of the possible role of the hypothalamus-pituitary-adrenal axis in CFS. For the baseline assessment, comparison will be made with cortisol concentrations in matched neighborhood controls. All patients will collect saliva for 2 consecutive days at four different time points (directly after awakening, 30 min after awakening, 11 a.m., 8 p.m.), using the passive drool method. Saliva will be stored at -80 °C until further analysis.
8. Microbiome determination in stools. This will give more insight into the microbial flora of the host, which is a new field of interest in a wide range of diseases. The availability of well-defined patients with CFS and matched controls is a great opportunity in an unexplored area of CFS research to assess whether the microbiome of CFS patients is peculiar. Patients will collect feces at home, and all samples will be stored at –80 °C until further analysis.

All secondary outcome measures will be assessed at baseline and directly following the intervention (Fig. 1). Only the VAS pain scale will be filled in weekly (together with the CIS) during the trial.

At 6 months, all questionnaires evaluating primary and secondary outcomes will be repeated to evaluate if the expected effects of the medication are maintained during the follow-up period of 5 months.

Other study parameters collected at baseline will include the following: demographic data, medical history, psychiatric history, serology results collected before inclusion in the study, use of medication, smoking and the use of alcohol and drugs.

### Withdrawal of individual participants

Subjects can leave the study at any time for any reason if they wish to do so without any consequences. The investigator or independent physician can decide to withdraw a subject from the study for urgent medical reasons. In general, noncompleters are not to be replaced. Subjects withdrawn from the study for a medical reason or adverse event will receive adequate follow-up. All analysis will be done according to the Intention-to-Treat principle (ITT). In case of discontinuation, efforts will be made to continue all study measurements. Withdrawn patients, or patients who are still severely fatigued following the intervention, will be offered regular care, which is CBT at the ECCF.

### Adverse events

Adverse events (AE) are defined as any undesirable experience occurring to a subject during the study, whether or not considered related to the investigational product. All adverse events reported spontaneously by the subject or observed by the investigator or his staff will be recorded. Anakinra is known as a very safe drug. Side effects are mainly related to irritation at the injection site, these reactions usually wane with prolonged treatment. We have reported from our own experience with long-term use of anakinra in patients with Schnitzler syndrome that the risk for infection is not enhanced, even in this elderly group [38]. Nevertheless, the package insert warns against infectious complications in patients with underlying illness. Rarely, neutropenia develops during treatment, which is a reason to discontinue therapy.

### Statistical analysis

The primary analysis will be the comparison between the two different treatment groups (anakinra or placebo) at 4 weeks. Data will be analyzed on an ITT basis. Missing values will be replaced using multiple imputation with fully conditional specification with at least five imputations. The imputation method that will be used is predictive mean matching for missing data in primary and secondary outcome measures. Aside from condition, we will use the following variables assessed at baseline to generate the imputations: duration of symptoms, age, BMI and baseline values of the outcome measures.

The results will be analyzed with SPSS for Windows. To determine if there is a significant difference between the intervention arm and placebo condition, ANCOVA will be used with the outcome measure on the second assessment as the dependent measure, the baseline score as the covariate, and condition as the fixed factor [39]. We will test if significant differences exist between both groups in mean age and BMI at baseline, both known to influence circulating cytokine levels. If so, these variables will be entered as covariates in the ANCOVA. When a statistically significant difference is found in the primary analysis, a sensitivity analysis will be performed on the basis of different assumptions about the values of missing data. For the secondary outcome measures, the same analysis will be repeated, but the secondary outcome measures at the second assessment will serve as the dependent variable and the scores at baseline as the covariate.

### Power calculation

The sample size is based on the fatigue subscale score of the CIS at 4 weeks. In the present study, the power calculation is based on results from comparable studies with CFS patients where fatigue severity was determined with the CIS-fatigue scale [40]. If interleukin-1 plays a central role in CFS symptomatology, we expect a considerable reduction of fatigue following treatment. Assuming a large controlled effect size of 0.85, an alpha of 0.05 and a power of 0.80, 23 patients are needed in each arm of the study. The number of patients can be further reduced by using ANCOVA, with the outcome measure on the second assessment as the dependent measure, the baseline score as the covariate, and condition as the fixed factor. In this kind of trials, ANCOVA yields greater power compared to other statistical methods [39]. Based on the correlation between the pre- and post CIS fatigue scale, the sample size of 23 can be multiplied by 0.883 (1 to 0.342^2^). Including an assumed dropout rate of 20 percent, we will have to include 25 patients in each group to demonstrate a significant difference between the medication group and placebo.

## Discussion

This study will be the first randomized placebo-controlled trial to evaluate the effect of blocking IL-1 on symptoms in patients with CFS. Earlier studies investigated cytokine production to be of relevance in CFS patients, but conflicting results have been published [11, 13]. A possible explanation is that good controls have not been used in these studies, and cytokines were measured in peripheral blood instead of in tissues.

Blinding the study for anakinra is a difficult procedure because of the occurrence of local skin reactions in a significant amount of patients. To maintain the double-blind design of this trial, side effects will be evaluated by an independent PA. In earlier “double blind” trials, medication was injected in the presence of the main investigator [27]. The effect of anakinra will be measured up to 6 months after the start of treatment. In this manner, we can evaluate the long-term effect of blocking IL-1 for a short period. It will provide more insight into the best treatment for CFS patients when blocking IL-1 appears to be effective.

In conclusion, this study will provide more insight into the pathophysiology and treatment of patients with CFS. If an effective treatment can be found, this will drastically improve the quality of life in patients with this disabling disease.

### Trial status

Recruiting.

## Abbreviations

AE: adverse event
BDI: Beck Depression Inventory
CBT: cognitive behavioral therapy
CFS: chronic fatigue syndrome
CIS: Checklist Individual Strength
ECCF: Expert Center Chronic Fatigue at Radboud University Medical Center
EudraCT: European Drug Regulatory Affairs Clinical Trials
GCP: good clinical practice
GET: gradual exercise therapy
HAM-D: Hamilton Rating Scale for Depression
HPA-axis: hypothalamic-pituitary-adrenal-axis
IL-1: interleukin-1
IL-1Ra: interleukin-1 receptor antagonist
IL-8: interleukin-8
MINI: The Mini-International Neuropsychiatric Interview
PA: Physician Assistant
SCL-90: Symptom Checklist 90
s.c.: subcutaneously
SD: standard deviation
SF-36: Short Form 36
SFQ: Shortened Fatigue Questionnaire
SIP: Sickness Impact Profile
TNFα: tumor necrosis factor α
VAS: Visual Analog Scale
